# Bilateral orthodontic extractions using physics versus conventional forceps among Indian patients

**DOI:** 10.6026/97320630019143

**Published:** 2023-01-31

**Authors:** Vidhi Sangra, HR Hemanth Kumar, Arundhati Singh, Sandeep Singh, Godana Akhilasiri, Vivek Agarwal

**Affiliations:** 1 Consultant Oral and Maxillofacial Surgeon at Pulse Hospital, Jammu, Jammu and Kashmir, India; 2 Department of Dentistry, Karwar Institute of Medical Sciences, Karwar, Karnataka, India; 3 Department of Oral and Maxillofacial Surgery, Hazaribagh College of Dental Sciences and Hospital, Hazaribagh, Jharkhand, India; 4 Department of Orthodontic and Dentofacial Orthopedics, Terna Dental College and Hospital, Navi Mumbai, Maharashtra, India; 5 BDS, Private Practitioner at Ravi Dental Clinic, Kadapa, Andhra Pradesh, India; 6 Department of Orthodontics and Dentofacial Orthopedics, Haldia Institute of Dental Sciences and Research, Haldia, West Bengal, India

**Keywords:** Conventional forceps, orthodontic extraction, premolar extraction, physics forceps

## Abstract

It is of interest to assess whether or not physics forceps are superior to traditional forceps for the extraction of premolar teeth in orthodontic procedures. Tooth and buccal bone fractures, as well as extraction time, lacerated gingiva, postoperative discomfort, and infection, were all measured in this research of both types of forceps extraction. Twenty individuals who need orthodontic extraction on both jaws were enrolled in the research. One arch's premolars were removed in two appointments, the first using Physics forceps and the second using conventional ones. The subsequent assignment included extraction from the obverse arch. Intraoperative evaluations included assessments of factors such as tooth and buccal bone fractures, surgical time, and gingival lacerations; postoperative assessments of pain and infection were conducted on days 1, 3, and 7. With physics forceps, the average time to remove a patient's mandible was 86.55 seconds, whereas traditional forceps required just 35.70 seconds. Using traditional forceps, the average pain score was 0.865 on day one after surgery, but with physics forceps, it was 3.30. The use of physics forceps resulted in one buccal bone fracture out of twenty premolar extractions. That so, no meaningful statistical change was seen. There was no tooth damage or post-operative infection with either set of forceps, it was found. Each forceps caused a Grade I laceration to the gingiva. The average time required removing a maxillary using physics forceps was 224.05 seconds, whereas the time required doing it with conventional forceps was 141.50 seconds. On a Visual Analogue Scale (VAS), the average first-day pain after surgery using physics forceps was 4.90, whereas using traditional forceps resulted in just 3.15. The difference between using physics forceps and regular forceps was statistically significant by the third postoperative day (2.05 vs 0.75). There was a statistically insignificant increase in the occurrence of buccal bone fracture and tooth fracture while using physics forceps. Both forceps and scissors caused just grade I lacerations, and there was no postoperative infection. These findings suggest that the use of physics forceps, as opposed to conventional forceps, may significantly lengthen the time required to remove orthodontic premolars on both sides of the mouth. Non-significant results were also found for additional criteria such as buccal bone fracture, tooth fracture, gingival laceration, and post-operative discomfort. When it comes to orthodontic premolar extraction, this research found that traditional forceps performed better than modern forceps across a range of measures, including intraoperative time and postoperative discomfort.

## Background:

Face is an index of the mind. Teeth play an important role in the facial esthetics, and also one"s personality. It also helps in functions like mastication and speech. Tooth can also be extracted for many reasons like irreversible dental pulpitis, advanced periodontal diseases and orthodontic purposes etc. Tooth extraction is considered as one of the most dreaded procedure by many people due to the unpleasant past experience of previous tooth extraction done for them or for others. [1] Tooth extraction, according to Geoffrey L. Howe, is the non-traumatic removal of a whole dental or tooth root with little stress to the investing tissues, ensuring a smooth recovery with no post-operative complications. Extractions may be split into two categories. They are closed extraction (intra-alveolar) or Transalveolar (open extraction). In closed extraction technique tooth is extracted using forceps and elevators by simple mechanical principles. In transalveolar extraction, muco periosteal flap is reflected and bone removal is done with burs or chisel and the tooth is removed. Transalveolar extraction is comparatively more traumatic than simple extraction and it takes more time for healing. [2] There are 3 mechanical principles for extraction. 1. Expansion of bony socket : In this, tooth is grasped by the forceps below the CEJ and specific tooth movements are given so that the roots of the tooth expands the alveolar socket followed by tearing of periodontal ligament fibres. The socket expansion depends on various factors like age, gender, occupation and anatomic location. 2. Lever and fulcrum principle: In this principle, an elevator is used for the removal of distal most teeth. The interdental bone serves as the fulcrum. Care should be taken not to keep the adjacent tooth as fulcrum to avoid adjacent tooth trauma. 3. Wedge principle: According to this principle, the instrument blade is wedged between tooth and socket wall to luxate the tooth to facilitate the extraction. [3-4] In closed extraction, various extraction forceps were introduced by Aristotle, who was the one who first described the mechanics of extraction forceps (384-322 BC). He described the mechanics 100 years before Archimides, who explained the principle of lever. During the period of 14-18th century an instrument called "Pellican" was used to extract teeth. During the early 20th century, modern forceps were invented for simple and atraumatic extraction. [5] For Atraumatic extraction, many instruments were introduced like Physics forceps, Benex extraction system, Periotome etc. Among this, physics forceps was introduced by "Goldent" in 2004 for atraumatic extraction purpose. [6] It is based on Class-I lever principle. It has a beak and bumper system. The beak is placed in the lingual/palatal surface of tooth below Cemento enamel Junction (CEJ) through gingival sulcus beneath the gingiva. The bumper is placed over the buccal soft tissue apical to the tooth. After proper placement, mild rotation force towards bumper side is given for some degrees without squeezing thehandle. Hence, there is a constant force exerted on the tooth which induces hyaluronic acid production and favors detachment of periodontal ligament fibers. After luxation, the tooth is removed using conventional forceps. There are various complications associated with traumatic tooth extraction. [7] The intraoperative complications are excessive bleeding, tooth fracture, buccal/lingual plate fracture, tuberocity fracture, injury to adjacent tooth, oro-antral communication, injury to soft tissue etc. Post-operative complications include delayed healing, infection, pain, trismus and dry socket. [8] Orthodontic extraction is mainly done in case of arch, tooth discrepancy which results in teeth crowding. In orthodontic extraction, great care should be given for atraumatic extraction as sufficient bone support is important for ideal tooth movement. Thus, buccal and lingual plates should be preserved with minimal socket expansion and result in less post-operative morbidity. This research compared the results of using physics forceps to those of using traditional forceps for extracting premolars for orthodontic purposes. The thickness of buccal plate and thin roots of premolar are crucial factors in determining the traumatic extraction of premolar. It is important to choose correct extraction system especially in orthodontic extraction. [9] Therefore, it is of interest to document the efficiency of physics versus conventional forceps in bilateral orthodontic extractions among Indian patients.

## Material and Methods:

The research was done at the Dental College and Hospital in the Oral and Maxillofacial Surgery division. Twenty individuals in total were recruited, and premolars from both arches were extracted on the first visit. Patients were given detailed information regarding the research and the surgery. Consents were gathered from participants in writing. Patients were requested to report on postoperative days 1, 3, and 7. Pain and postoperative infection were assessed on postoperative days one, three, and seven, and intraoperative outcomes included the rate of buccal bone fracture, tooth fracture, time to repair, and gingival laceration.

## Inclusion criteria:

[1] Bilateral extraction of upper and lower premolars for orthodontic treatment purpose.

[2] Age: 12-25 Years.

## Exclusion criteria:

[1] Mobile teeth.

[2] Carious teeth.

[3] Mal aligned teeth.

[4] Teeth indicated for trans-osseous extraction.

[5] Medically compromised patients.

## Armamentarium used:

[1] 2% lignocaine with 1:80000 adrenaline

[2] Molt No.9 periosteal elevator.

[3] Williams probe.

[4] Physics forceps

a) Upper right forceps (Upper right premolars)

b) Upper left forceps (Upper left premolars)

c) Lower universal forceps (Lower premolars)

[5] Conventional forceps

a) Upper premolar forceps

b) Lower premolar forceps

[6] Cotton gauze.

## Study design:

[1] It was a split mouth Study 20 Patients was included in the study that required bilateral extraction of premolars for orthodontic purpose.

[2] A number between (1 to 20) was given to each patient randomly by taking a lot. For Odd numbers Right side Extraction would be done with Conventional forceps and Left side with Physics Forceps. For even numbers Vice versa.

[3] Two premolars from the same arch were extracted at the first session; one set were removed using Physics forceps, while the other set were removed with traditional dental forceps. The subsequent assignment included extraction from the obverse arch.

[4] A variety of intra- and post-operative variables, including buccal bone fracture, tooth fracture, time required, and gingival laceration, were assessed.

[5] Initial, 3-day, and 7-day postoperative assessments were performed.

## Procedure:

[1] There was an OPG or IOPA done before the surgery.

[2] For local anesthesia, a mixture of 1% lidocaine hydrochloride and 1% adrenaline was utilized.

[3] In extractions performed with standard forceps, the mucoperiosteal flap was merely reflected.

[4] The bumper of the forceps is positioned at the mucogingival junction perpendicular to the root, and the beak is anchored to the lingual or palatal surface, providing a stable grip.

[5] Without pressing the handle or moving the arm, a constant and gradual rotating force was delivered in the direction of bumper.

[6] Time was measured from when the beaks were placed on the tooth until the tooth was delivered from the socket.

## Evaluation criteria:

[1] Buccal bone fracture and tooth fracture were assessed clinically

[2] Time taken was measured using stopwatch. The interval between applications of forceps beaks in the tooth to delivery of tooth is calculated as the time taken for extraction.

[3] Gingival laceration was calculated using the following scale:

Postoperative Pain was calculated in the 1st, 3rd and 7th Post-operative days. It was measured using Visual Analogue Scale (VAS)

## Results:

**Figure 1:**
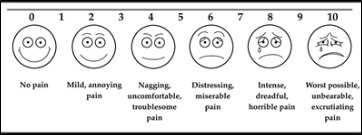
Post-operative infection was also assessed in the 1st, 3rd and 7th Postoperative days. Swelling, redness, pus discharge, pain were considered as infection.

**Figure 2:**
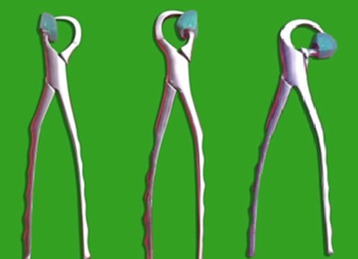
Lower Universal Physics forceps

The average amount of time needed to remove a patient's mandible using physics forceps was 86.55 seconds, whereas using conventional forceps only took 35.70 seconds. On a Visual Analogue Scale (VAS), the average pain experienced by patients on the first postoperative day was 3.30 with physics forceps and 0.865 with traditional forceps. Just one of twenty premolar extractions with physics forceps resulted in a buccal bone fracture. However, statistically speaking, it was not significant. No tooth damage or postoperative infection has been seen in either of the forceps. Both forceps caused just little bleeding at the gingival margin (grade I). Using physics forceps for maxillary extraction took an average of 224.05 seconds, whereas using traditional forceps took an average of 141.50 seconds. On the first postoperative day, the average VAS score for pain was 4.90 with physics forceps and 3.15 with traditional forceps. On the third postoperative day, the difference between using physics forceps and using regular forceps was statistically significant (2.05 vs. 0.75). The increased risk of buccal bone fracture and tooth fracture during physics forceps extraction was not statistically significant. Both forceps were shown to cause gingival laceration of grade I severity. No patients in either group developed an infection after surgery. There was no tooth damage or postoperative infection after using either set of mandibular premolar extraction forceps, it was found. Both forceps have produced Grade I gingival laceration. Only one out of twenty premolars extracted using physics forceps resulted in a fractured buccal bone. However, statistical analysis indicates that this variance is not significant (p=0.311). The mean time taken to extract the premolar by physics forceps has been86.55secs with the standard deviation of 32.4secs. Similarly for the conventional forceps the mean time taken has been 35.7secs with the standard deviation of 8secs.The pain level has been assessed using visual analogue scale of 0-10. The mean pain level at post-operative day1 has been mild level in both the forceps and gradually reduces. At the end of post-operative day 7, it has been observed no pain for both the forceps. The significant p-value of the between comparison indicates in general the pain level has been higher in the physics forceps compared to conventional forceps. The "Day-assessment" p-value reveals that irrespective of the forceps has been adopted; the pain level has been reduced from day 1 to day7. Further "Repeated Contrast test" shown in Table 1, Table 2 ensures from day 1 to day3 the pain level has been significantly reduced for both the forceps used and similarly from day3 to day7 also the pain level has been reduced. (Figure 1, Figure 2)

## Discussion:

Extracting a tooth is a minor surgical treatment that should be carefully planned and executed with the utmost care and precision to avoid causing any unnecessary damage to the patient. An ideal tooth extraction, according to Geoffrey L. Howe, is the painless removal of the whole tooth or tooth root with minimum stress to the investing tissues, allowing for a smooth wound healing process and no post-operative complications. Orthodontic extraction is mainly done in case of arch tooth discrepancy which results in crowding. In orthodontic extraction great care should be given for atraumatic extraction as sufficient bone support is important for ideal tooth movement. So buccal and lingual plates should be preserved with minimal socket expansion and thus result in less post-operative morbidity. This research compared the success rates of using physics forceps vs traditional forceps for the extraction of bilateral orthodontic premolars. Buccal bone fracture, tooth fracture, treatment delay, gingival laceration, postoperative discomfort, and postoperative infection were among the factors studied. Six steps make up the process of using the Physics Forceps: First, the gingival attachment is dislodged from the tooth (with the aid of a periosteal elevator, such as the Molt No. 9), then the "beak" is placed on a secure palatal/lingual application point (root surface), and finally the "bumper" (with a single-use plastic sleeve attached) is placed on the buccal aspect of the alveolus at the level of the mucogingival junction (this acts as Five, the tooth is lifted occlusally a few millimeters from its socket; six, the tooth is delivered using a haemostat, rongeurs, or standard forceps. Choi [10] emphasized that even multi-rooted molars may be removed securely without fracture utilizing the physics forceps, and hence ASE should be regarded as a dependable extraction approach for safe and effective intentional replantation (IR). According to Khaled K [11], By applying pressure uniformly over the bone's surface with the wrist alone, physics forceps reduce the frequency with which the buccal bone is broken. When placed on the alveolar ridge of the bucx, the bumper exerts a compressive stress on the buccal bone, keeping it in its proper location. This result was in agreement with the result of Kosinski [12] who found that physics forceps' buccal movement was too slow and insufficient to fracture the buccal bone plate, leading them to the conclusion that the best results could be seen in the immediately post-operative phase when implants were placed right after teeth were extracted using the same method. In our study, buccal bone fracture and tooth fracture in physics group was higher than the conventional group in premolar extraction. In mandible, among 20 cases buccal bone fracture occurred in 1 case in physics group and no buccal bone fracture occurred in conventional group. The maxillary buccal bone fractured in 4 instances in the physics group and 1 case in the traditional group. None of the participants in either group had a broken mandible due to a tooth. Four people in the physics group and two people in the conventional group had a tooth fracture in the maxilla. Our research found that the use of physics forceps to remove premolars was more time-consuming than using traditional forceps. The average amount of time it takes to remove a mandibular premolar with physics forceps is 86.55 seconds, whereas using conventional forceps only takes 35.70 seconds. Time required for maxillary premolar extraction using physics forceps was 224.05 seconds, whereas using conventional forceps took 141.50 seconds.It is not in accordance with Mandal S et al. [13], in their comparative study they reported that the mean extractiontime of 139.8 sec using physics forceps and 236 sec using conventional forceps, whereas S Hariharan et al. [14], Mean extraction time utilizing physics forceps was 29.4sec and traditional forceps was 43.5sec, with no statistically significant difference found. Due to the mechanical advantage of the physics forceps, applying greater power to the buccal side would result in buccal bone and tooth breakage, which is why our research required longer time for extraction. So, more time is needed to complete the extraction. Tooth luxation is the result of the continual pressure applied by the physics forceps, which triggers the release of the Hyaluronidase enzyme and the subsequent destruction of the periodontal ligament fibers. According to Dym and Weiss [15], when using the Physics forceps for extraction, there is no need to utilize an elevator or lift the mucoperiosteal flap beforehand. This is especially helpful when an atraumatic extraction is necessary.Saumen mandel [16] using traditional forceps was associated with a higher risk of gingival laceration, as revealed in his research. Sonune Avinash M [17], in his research, he found that any kind of forceps may be used to retrace the gums without causing a laceration. As a result of reduced damage to soft and hard tissues, physics forceps resulted in less blood loss during extraction. Harsh S Patel [18] According to the results of his research, utilizing physics forceps resulted in far smaller changes in gingival level before and after extraction than using conventional forceps, suggesting that the latter method was more damaging to the gingival tissues. Mandal S et al. [19], clinically examined for the presence or absence of laceration on marginal gingiva to compare the state of gingival tissue after extraction utilizing physics forceps and conventional forceps. They determined that extractions performed using physics forceps were less traumatic than those performed with conventional forceps since only 16.6% of patients in the physics forceps group had gingival laceration. In our study we graded the gingival laceration as grade I (0-5mm), grade II (5-10mm), grade III (>10mm) and grade IV (torn gingiva). [19] All gingival lacerations seen in our investigation were classified as grade I. When comparing the laceration caused by physics forceps to that caused by regular forceps, there was no discernible difference. Physics forceps have the benefit of not requiring mucoperiosteal reflection in order to properly set the beak. Pain after physics forceps vs. traditional forceps extractions was assessed by Harsh S. Patel using the Visual Analogue Scale (VAS). [13] In his research, he found no statistically significant difference in VAS score between the two groups on the first and third postoperative days. However, a lower mean VAS score was noted in the physics forceps group, which may be attributable to the relatively less traumatic extractions performed by physics forceps. These findings are consistent with those of Hariharan et al. [1414], who also discovered that the physics forceps group had much less postoperative discomfort on the first postoperative day than the traditional forceps group. The increased pain in the physics group is due to 2 reasons. One is the increased intraoperative time for physics forceps comparing to the conventional forceps. Increased intraoperative time causes more tissue trauma and leads to more pain. The other reason for increased pain in the physics group is due to the compression of the bumper in buccal soft tissue. Many patients reported with pain in the site of bumper application rather than the extracted socket. Avinash [17] found no statistically significant differences in soft tissue healing between the physics forceps and traditional forceps groups on days 7, 14, and 21 post-operatively, although he did highlight that healing is impacted by a variety of local and systemic variables. We found no cases of postoperative infection in either group. On day seven after surgery,there was no difference in the rate of infection or the rate at which patients recovered from surgery.

## Conclusion:

This research found that the use of physics forceps for bilateral orthodontic premolar extraction significantly increased the time required and the post-operative discomfort compared to the use of conventional forceps. The 1st day post-operative pain was more in physics forceps extracted socket. The result also showed that, the pain subsided gradually after 1 week on both forceps extracted sockets. Even though, buccal bone fracture and tooth fracture were present there was no statistical significance. The gingival laceration was grade 1 in both groups for all cases. There was no postoperative infection in both groups. Based on measures of intraoperative time and patient comfort experienced immediately after surgery, the authors of this research suggest that conventional forceps are preferable than physics forceps for orthodontic premolar extraction.

## Figures and Tables

**Table 1:** A repeated contrast test result

Source	Comparison	F-value	P-value
Day	Day1vsDay3	377.19	0
	Day3vsDay7	12.981	0.001
Day*Forceps	Day1vsDay3	21	0
	Day3vsDay7	1.123	0.296

**Table 2:** Comparison between mandible and maxilla on pain level by forceps

Site	Forceps	Day 1		Day 3		Day 7	
		Mean	SD	Mean	SD	Mean	SD
Mandible	Physics forceps	3.3	0.865	0.55	0.826	0	0
	Conventional	2	0.562	0.3	0.657	0	0
Maxilla	Physics forceps	4.9	0.788	2.05	0.605	0	0
	Conventional	3.15	0.875	0.75	0.786	0	0
The "Between Comparisons" of the interaction effect "Forceps *site" clearly infers that pain level experienced by the participants has been higher for physics forceps at the maxilla site.
